# Poly[guanidinium [tri-μ-formato-κ^6^ *O*:*O*′-formato-κ^2^ *O*,*O*′-yttrium(III)]]

**DOI:** 10.1107/S1600536814013440

**Published:** 2014-06-14

**Authors:** Mark A. Rodriguez, Dorina F. Sava Gallis, Tina M. Nenoff

**Affiliations:** a PO Box 5800, MS 1411, Sandia National Laboratories, Albuquerque, NM 87185-1411, USA

## Abstract

In the title coordination polymer, {(CH_6_N_3_)[Y(CHO_2_)_4_]}_*n*_, the yttrium(III) ion is coordinated by one *O*,*O*-bidentate formate ion and six μ_2_ bridging formate ions, generating a square-anti­prismatic YO_8_ coordination polyhedron. The bridging formate ions connect the metal ions into an anionic, three-dimensional network. Charge compensation is provided by guanidinium ions, which inter­act with the framework by way of N—H⋯O hydrogen bonds. The guanidine molecules reside in porous channels of 3.612 by 8.189 Å, when considering the van der Waals radii of the nearest atoms (looking down the *a*-axis).

## Related literature   

Liu *et al.* (2011 ▶) have published the erbium (Er) analog of the title compound, *catena*-(tris­(μ-formato)-formato-erbium di­amino­methaniminium , with nearly identical cell parameters and unit-cell volume. They also document a similar Er-based structure that employs a different solvent (1*H*-imidazol-3-ium). The presence of formic acid in the reaction is likely a result of the DMF hydrolysis as it is known to be a common impurity in DMF (IUPAC, 1977 ▶). The di­amino­methaniminium ion was generated *in situ*, through 2-amino-4,6-di­hydroxy­pyrimidine ring cleavage (Calza, *et al.*, 2004 ▶). In regard to the observed chirality of the title compound, it has been previously documented that there is a great propensity for virtually any metal-organic framework (MOF) to crystallize in a chiral space group (Lin, 2007 ▶). 
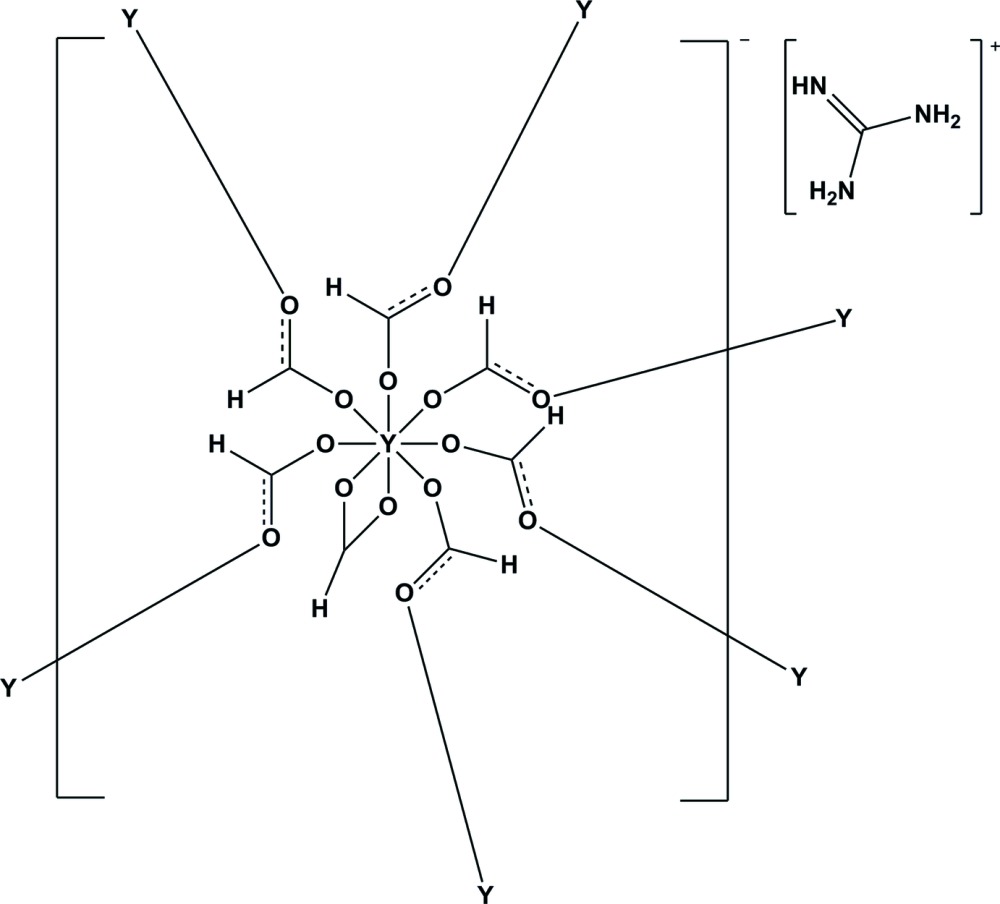


## Experimental   

### 

#### Crystal data  

(CH_6_N_3_)[Y(CHO_2_)_4_]
*M* *_r_* = 329.07
Orthorhombic, 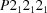

*a* = 6.6537 (13) Å
*b* = 8.0998 (15) Å
*c* = 20.179 (4) Å
*V* = 1087.5 (4) Å^3^
*Z* = 4
Mo *K*α radiation
μ = 5.40 mm^−1^
*T* = 188 K
0.35 × 0.15 × 0.12 mm

#### Data collection  

Bruker APEX CCD diffractometer
Absorption correction: multi-scan (*SADABS*; Bruker, 2005 ▶) *T*_min_ = 0.247, *T*_max_ = 0.541
8974 measured reflections
2428 independent reflections
2219 reflections with *I* > 2σ(*I*)
*R*_int_ = 0.021

#### Refinement  

*R*[*F* ^2^ > 2σ(*F* ^2^)] = 0.017
*wR*(*F* ^2^) = 0.036
*S* = 0.92
2428 reflections
154 parameters
H-atom parameters constrained
Δρ_max_ = 0.35 e Å^−3^
Δρ_min_ = −0.25 e Å^−3^
Absolute structure: Flack *x* determined using 835 quotients [(*I* ^+^)−(*I* ^−^)]/[(*I* ^+^)+(*I* ^−^)] (Parsons & Flack, 2004 ▶)
Absolute structure parameter: 0.000 (4)

### 

Data collection: *APEX2* (Bruker, 2005 ▶); cell refinement: *SAINT* (Bruker, 2005 ▶); data reduction: *SAINT-Plus* (Bruker, 2005 ▶); program(s) used to solve structure: *SHELXTL* (Sheldrick, 2008 ▶); program(s) used to refine structure: *SHELXTL*; molecular graphics: *MaterialsStudio* (Accelrys, 2013 ▶); software used to prepare material for publication: *SHELXTL*.

## Supplementary Material

Crystal structure: contains datablock(s) I. DOI: 10.1107/S1600536814013440/bv2234sup1.cif

Structure factors: contains datablock(s) I. DOI: 10.1107/S1600536814013440/bv2234Isup2.hkl

CCDC reference: 1007433

Additional supporting information:  crystallographic information; 3D view; checkCIF report

## Figures and Tables

**Fig. 1.**
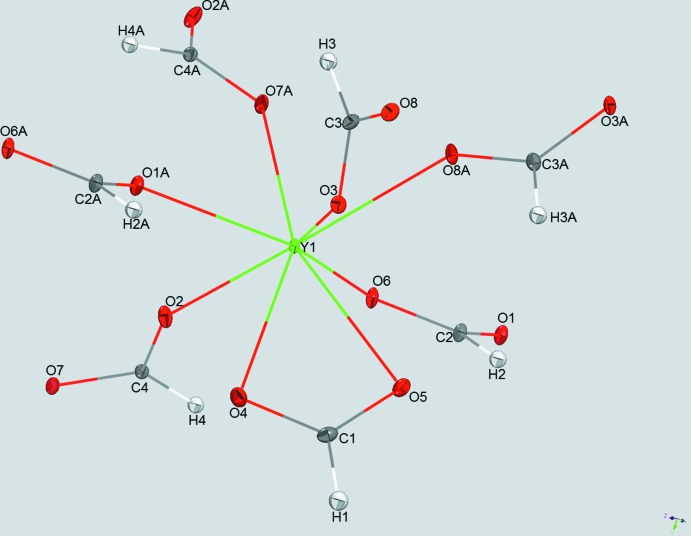
The molecular structure of Y metal center for compound (I), with atom labels and 50% probability displacement ellipsoids for non-H atoms.

**Fig. 2.**
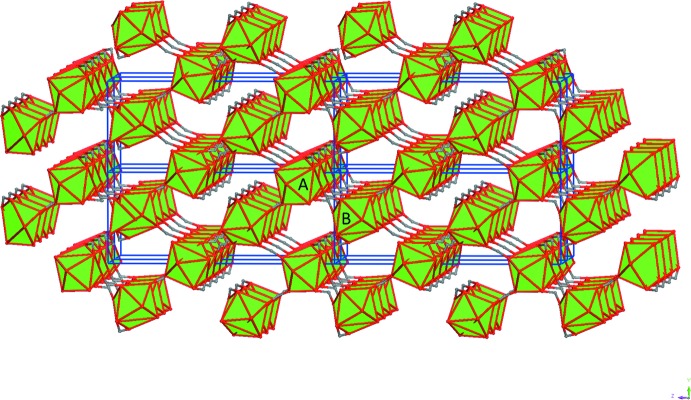
Packing diagram of (I) with multiple unit cells to show connectivity and solvent void locations. For clarity, H-atoms have been removed from formate ligands. Clusters A and B are labeled for reference purposes. See text for details.

**Fig. 3.**
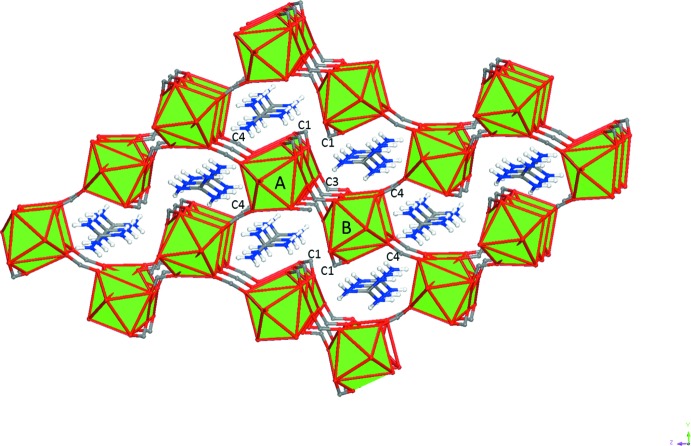
Zoomed packing diagram for (I) showing location of the diaminomethaniminium molecules in the void space. Additionally, formate molecules are labeled by their respective C atoms to illustrate how the formate molecules bridge the Y-based clusters. A and B labels are given to identify two of the metal clusters (or bi-cluster) bridged by the C3 formate ligand. A and B clusters reference back to Figure 2 to orient the viewer to the zoomed region of the packing diagram. H atoms have been removed from formate ligands for clarity, whereas H-atoms are present on diaminomethaniminium solvent molecules. See text for details.

## supplementary crystallographic information

### S1. Comment

The title compound (I) C_4_H_4_O_8_Y . (CH_6_N_3_) is comprised of a yttrium (Y) atom fully coordinated by formate molecules, thereby creating a three-dimensional network structure of linked Y formate nodes. Figure 1 shows the coordination of the formate groups about Y atom. The figure shows three monodentate (bridging) formate molecules designated here by their associated C atoms (C2, C3, C4) along with a bidentate formate (C1) molecule exclusively bound to the metal center. The monodentate formate molecules bridge to neighboring metal clusters to generate the three-dimensional network. Considering the summation of charge around the Y^+3^ atom, one obtains a full -1 charge from the bidentate molecule along with a total of six monodentate formate molecules, each contributing -0.5 worth of charge for a total of -4 charge for each Y-formate cluster. This net -1 charge on the cluster is balanced by the presence of one diaminomethaniminium molecule for every cluster present. Figure 2 shows the three-dimensional network of the structure as viewed down the *a* axis. The plot shows Y-formate clusters as node polyhedra that are linked by formate ligands. For clarity purposes, hydrogen atoms on formate molecules as well as the diaminomethaniminium solvent molecules have been removed. The void space between the linked nodes are filled by the diaminomethaniminium ions which pack along the *a* axis direction. Two of the Y-formate polyhedra in Figure 2 are labeled as A and B clusters and are located near the center of the image. This has been done for reference purposes.

Figure 3 shows a smaller region of the three-dimensional network to better discuss connectivity. This figure, also viewed down the *a* axis, shows the location of the diaminomethaniminium molecules within the pores of the framework. Noting the polyhedra labeled A and B in Figure 3, one can compare this area back to Figure 2 and how it relates to the larger three-dimensional array. Considering the lattice shown in Figure 3, one can see that the A—B polyhedra pair (or bi-cluster) are linked by the C3 formate ligand, which exclusively bridges the A and B nodes. This A—B polyhedral bi-cluster is then bridged to neighboring A—B bi-clusters *via* the C4 formate ligand along the b-c plane (*i.e.* parallel to the plane of the image). The C1 formate is the bidentate ligand and does not link to neighboring Y-formate nodes, but instead truncates within the void space. Note that the formate ligands are plotted in Figure 3 without bound H atoms for purposes of clarity, while the diaminomethaniminium molecules are shown with H atoms present. The C2 formate ligand is not visible in this image, but links the Y-formate nodes along the *a* axis direction. It is worth noting in regard to the C2 formate molecule that when one considers the location of the C2 and C2A formate molecules in Figure 1; it is clear that these formate ligands (labeled as O6A—C2A—O1A and O6—C2—O1) are on nearly opposite sides of the Y metal center and thus can link the Y-formate nodes in a continuous fashion along the *a* axis. The O1A—Y—O6 angle is 141.34 (6)° which indicates the near opposing locations of the C2 and C2A formate ligands. This opposing orientation of the paired formate ligands does not hold true for the other formate molecules. Considering the C3 and C3A ligands, these two ligands are related through the Y metal center by the O3—Y—O8A bond angle of 74.03 (6)°. Likewise, the C4 and C4A formate ligands are related by the O2—Y1—O7A bond angle of 83.47 (6)°. In both of these cases the O—Y—O bond is close to 90° which serves to facilitate a zigzag bonding array of connectivity between adjacent Y nodes. In this arrangement the C3 formate molecules bridge the A—B bi-cluster by alternating orientation along the *a* axis direction; whereas, the C4 formate alternates orientation along the *b* axis direction in a similar zigzag fashion, as can be assessed by careful evaluation of Figure 3.

In regard to the observed chirality of (I), it has been previously documented that there is a great propensity for virtually any Metal-Organic Framework (MOF) to crystallize in a chiral space group (Lin, 2007). This is thought to be inherent to the topological variety of these materials, as there are a multitude of coordination capabilities between the metal nodes and organic ligands.

### S2. Experimental

The reaction mixture containing Y(NO_3_)_3_^.^ 6H_2_O (0.0166 g, 0.0433 mmol), and 2-amino-4,6-DHPm (2-amino-4,6-dihydroxypyrimidine, 0.0165 g, 0.1298 mmol) in 2 ml of *N*,*N*'-dimethylformamide (DMF) was placed in a convection oven at 90°C for 18 h, followed by subsequent heating at 115°C for 24 h. The presence of formic acid in the reaction is likely a result of the DMF hydrolysis as it is known to be a common impurity in DMF (IUPAC, 1977). The diaminomethaniminium ion was generated *in situ*, through 2-amino-4,6-dihydroxypyrimidine ring cleavage (Calza, *et al.*, 2004).

### Crystal data

**Table:**

(CH_6_N_3_)[Y(CHO_2_)_4_]	*D*_x_ = 2.010 Mg m^−^^3^
*M**_r_* = 329.07	Mo *K*α radiation, λ = 0.71073 Å
Orthorhombic, *P*2_1_2_1_2_1_	Cell parameters from 200 reflections
*a* = 6.6537 (13) Å	θ = 1.0–25.0°
*b* = 8.0998 (15) Å	µ = 5.40 mm^−^^1^
*c* = 20.179 (4) Å	*T* = 188 K
*V* = 1087.5 (4) Å^3^	Tabular, colorless
*Z* = 4	0.35 × 0.15 × 0.12 mm
*F*(000) = 656	

### Data collection

**Table:**

Bruker APEX CCD diffractometer	2219 reflections with *I* > 2σ(*I*)
Radiation source: fine-focus sealed tube	*R*_int_ = 0.021
ω and φ scans	θ_max_ = 27.5°, θ_min_ = 2.0°
Absorption correction: multi-scan (*SADABS*; Bruker, 2005)	*h* = −8→8
*T*_min_ = 0.247, *T*_max_ = 0.541	*k* = −10→10
8974 measured reflections	*l* = −26→26
2428 independent reflections	

### Refinement

**Table:**

Refinement on *F*^2^	Secondary atom site location: difference Fourier map
Least-squares matrix: full	Hydrogen site location: inferred from neighbouring sites
*R*[*F*^2^ > 2σ(*F*^2^)] = 0.017	H-atom parameters constrained
*wR*(*F*^2^) = 0.036	*w* = 1/[σ^2^(*F*_o_^2^) + (0.0265*P*)^2^] where *P* = (*F*_o_^2^ + 2*F*_c_^2^)/3
*S* = 0.92	(Δ/σ)_max_ = 0.001
2428 reflections	Δρ_max_ = 0.35 e Å^−^^3^
154 parameters	Δρ_min_ = −0.25 e Å^−^^3^
0 restraints	Absolute structure: Flack *x* determined using 835 quotients [(*I*^+^)-(*I*^-^)]/[(*I*^+^)+(*I*^-^)] (Parsons & Flack, 2004)
Primary atom site location: structure-invariant direct methods	Absolute structure parameter: 0.000 (4)

### Special details

**Table:**

Geometry. All e.s.d.'s (except the e.s.d. in the dihedral angle between two l.s. planes) are estimated using the full covariance matrix. The cell e.s.d.'s are taken into account individually in the estimation of e.s.d.'s in distances, angles and torsion angles; correlations between e.s.d.'s in cell parameters are only used when they are defined by crystal symmetry. An approximate (isotropic) treatment of cell e.s.d.'s is used for estimating e.s.d.'s involving l.s. planes.

### Fractional atomic coordinates and isotropic or equivalent isotropic displacement parameters (Å^2^)

**Table:**

	*x*	*y*	*z*	*U*_iso_*/*U*_eq_	
Y1	0.50681 (4)	0.94595 (3)	0.11947 (2)	0.01148 (7)	
O1	0.1701 (3)	0.9359 (3)	0.15590 (10)	0.0207 (5)	
O2	0.4894 (3)	1.0890 (2)	0.21831 (8)	0.0248 (4)	
O3	0.3325 (3)	0.9006 (2)	0.01947 (9)	0.0200 (5)	
O4	0.4007 (3)	1.2229 (3)	0.09013 (10)	0.0277 (5)	
O5	0.6744 (3)	1.1283 (3)	0.04277 (10)	0.0238 (5)	
O6	0.8387 (3)	0.9575 (3)	0.16045 (9)	0.0197 (5)	
O7	0.4976 (4)	0.7212 (2)	0.18650 (8)	0.0222 (4)	
O8	0.6868 (3)	0.7345 (3)	0.06301 (9)	0.0223 (5)	
C1	0.5499 (4)	1.2410 (4)	0.05256 (14)	0.0250 (7)	
H1	0.5684	1.3439	0.0309	0.030*	
C2	1.0038 (4)	0.9690 (3)	0.13083 (11)	0.0192 (5)	
H2	1.0020	1.0056	0.0861	0.023*	
C3	0.7450 (4)	0.7212 (4)	0.00448 (16)	0.0205 (7)	
H3	0.7204	0.8119	−0.0242	0.025*	
C4	0.4192 (4)	0.6671 (3)	0.23804 (13)	0.0176 (6)	
H4	0.2799	0.6869	0.2445	0.021*	
C5	0.9892 (5)	0.9309 (3)	0.33693 (12)	0.0188 (5)	
N1	0.9596 (3)	0.9298 (3)	0.40184 (11)	0.0277 (6)	
H1A	1.0410	0.9851	0.4281	0.033*	
H1B	0.8585	0.8739	0.4187	0.033*	
N2	1.1401 (4)	1.0142 (3)	0.31080 (13)	0.0312 (7)	
H2A	1.1587	1.0136	0.2676	0.037*	
H2B	1.2221	1.0703	0.3365	0.037*	
N3	0.8665 (4)	0.8468 (3)	0.29787 (12)	0.0248 (6)	
H3A	0.8857	0.8467	0.2547	0.030*	
H3B	0.7656	0.7911	0.3150	0.030*	

### Atomic displacement parameters (Å^2^)

**Table:**

	*U*^11^	*U*^22^	*U*^33^	*U*^12^	*U*^13^	*U*^23^
Y1	0.00867 (10)	0.01734 (11)	0.00843 (10)	0.00127 (16)	−0.00014 (14)	−0.00069 (10)
O1	0.0101 (9)	0.0329 (13)	0.0189 (11)	0.0016 (11)	−0.0001 (8)	0.0014 (12)
O2	0.0161 (9)	0.0379 (11)	0.0204 (9)	0.0013 (13)	−0.0019 (11)	−0.0129 (8)
O3	0.0180 (10)	0.0264 (12)	0.0155 (10)	−0.0032 (8)	−0.0031 (8)	−0.0061 (9)
O4	0.0339 (12)	0.0259 (12)	0.0233 (12)	0.0090 (10)	0.0053 (10)	0.0032 (10)
O5	0.0183 (11)	0.0287 (12)	0.0245 (12)	0.0003 (9)	0.0003 (9)	0.0077 (10)
O6	0.0112 (10)	0.0342 (13)	0.0137 (11)	0.0017 (11)	0.0008 (8)	−0.0009 (11)
O7	0.0227 (9)	0.0273 (9)	0.0167 (9)	0.0074 (13)	0.0068 (12)	0.0089 (7)
O8	0.0245 (11)	0.0308 (12)	0.0117 (10)	0.0083 (10)	0.0038 (8)	−0.0026 (9)
C1	0.030 (2)	0.0229 (15)	0.0219 (16)	−0.0066 (13)	−0.0048 (12)	0.0054 (13)
C2	0.0142 (12)	0.0292 (14)	0.0142 (13)	−0.0027 (18)	0.0005 (15)	−0.0015 (10)
C3	0.0196 (14)	0.023 (2)	0.0188 (15)	0.0060 (14)	−0.0002 (11)	0.0022 (15)
C4	0.0156 (13)	0.0181 (14)	0.0191 (15)	0.0005 (11)	0.0006 (11)	0.0009 (13)
C5	0.0186 (13)	0.0184 (12)	0.0195 (12)	−0.0011 (19)	−0.0006 (14)	0.0002 (10)
N1	0.0280 (16)	0.0385 (14)	0.0167 (11)	−0.0086 (13)	−0.0013 (10)	−0.0019 (11)
N2	0.0265 (15)	0.0379 (16)	0.0293 (16)	−0.0136 (12)	0.0043 (13)	−0.0033 (13)
N3	0.0290 (14)	0.0303 (15)	0.0150 (12)	−0.0137 (12)	0.0008 (11)	0.0027 (11)

### Geometric parameters (Å, º)

**Table:**

Y1—O7	2.2688 (16)	C1—H1	0.9500
Y1—O2	2.3095 (16)	C2—O1^iv^	1.246 (3)
Y1—O3	2.3562 (18)	C2—H2	0.9500
Y1—O1	2.3593 (19)	C3—O3^v^	1.244 (4)
Y1—O6	2.3599 (18)	C3—H3	0.9500
Y1—O8	2.3803 (19)	C4—O2^vi^	1.243 (3)
Y1—O5	2.4127 (19)	C4—H4	0.9500
Y1—O4	2.425 (2)	C5—N2	1.320 (4)
Y1—C1	2.759 (3)	C5—N3	1.323 (3)
O1—C2^i^	1.246 (3)	C5—N1	1.324 (3)
O2—C4^ii^	1.243 (3)	N1—H1A	0.8800
O3—C3^iii^	1.244 (4)	N1—H1B	0.8800
O4—C1	1.257 (3)	N2—H2A	0.8800
O5—C1	1.248 (3)	N2—H2B	0.8800
O6—C2	1.254 (3)	N3—H3A	0.8800
O7—C4	1.243 (3)	N3—H3B	0.8800
O8—C3	1.248 (3)		
			
O7—Y1—O2	83.47 (6)	C2^i^—O1—Y1	135.17 (17)
O7—Y1—O3	111.85 (7)	C4^ii^—O2—Y1	147.4 (2)
O2—Y1—O3	142.49 (7)	C3^iii^—O3—Y1	133.39 (19)
O7—Y1—O1	76.16 (8)	C1—O4—Y1	91.45 (18)
O2—Y1—O1	72.59 (8)	C1—O5—Y1	92.25 (17)
O3—Y1—O1	78.12 (7)	C2—O6—Y1	130.97 (17)
O7—Y1—O6	81.26 (8)	C4—O7—Y1	142.44 (18)
O2—Y1—O6	74.00 (7)	C3—O8—Y1	132.2 (2)
O3—Y1—O6	140.07 (7)	O5—C1—O4	122.3 (3)
O1—Y1—O6	141.33 (6)	O5—C1—Y1	60.88 (15)
O7—Y1—O8	73.83 (6)	O4—C1—Y1	61.45 (15)
O2—Y1—O8	143.08 (7)	O5—C1—H1	118.9
O3—Y1—O8	74.07 (6)	O4—C1—H1	118.9
O1—Y1—O8	127.01 (7)	Y1—C1—H1	177.7
O6—Y1—O8	74.06 (7)	O1^iv^—C2—O6	124.6 (2)
O7—Y1—O5	152.40 (7)	O1^iv^—C2—H2	117.7
O2—Y1—O5	105.66 (7)	O6—C2—H2	117.7
O3—Y1—O5	76.91 (7)	O3^v^—C3—O8	125.6 (3)
O1—Y1—O5	131.30 (7)	O3^v^—C3—H3	117.2
O6—Y1—O5	76.59 (7)	O8—C3—H3	117.2
O8—Y1—O5	84.32 (7)	O2^vi^—C4—O7	124.5 (3)
O7—Y1—O4	151.64 (7)	O2^vi^—C4—H4	117.7
O2—Y1—O4	74.47 (7)	O7—C4—H4	117.7
O3—Y1—O4	77.99 (7)	N2—C5—N3	119.6 (2)
O1—Y1—O4	80.31 (8)	N2—C5—N1	120.8 (3)
O6—Y1—O4	108.74 (8)	N3—C5—N1	119.6 (3)
O8—Y1—O4	134.04 (7)	C5—N1—H1A	120.0
O5—Y1—O4	53.95 (7)	C5—N1—H1B	120.0
O7—Y1—C1	171.61 (8)	H1A—N1—H1B	120.0
O2—Y1—C1	89.62 (8)	C5—N2—H2A	120.0
O3—Y1—C1	76.53 (8)	C5—N2—H2B	120.0
O1—Y1—C1	106.33 (8)	H2A—N2—H2B	120.0
O6—Y1—C1	92.29 (8)	C5—N3—H3A	120.0
O8—Y1—C1	109.67 (8)	C5—N3—H3B	120.0
O5—Y1—C1	26.86 (7)	H3A—N3—H3B	120.0
O4—Y1—C1	27.10 (7)		

Symmetry codes: (i) *x*−1, *y*, *z*; (ii) −*x*+1, *y*+1/2, −*z*+1/2; (iii) *x*−1/2, −*y*+3/2, −*z*; (iv) *x*+1, *y*, *z*; (v) *x*+1/2, −*y*+3/2, −*z*; (vi) −*x*+1, *y*−1/2, −*z*+1/2.
